# Determinants of tetanus toxoid immunization among pregnant women in Somaliland: evidence from the 2020 nationwide survey using a zero-inflated negative binomial model

**DOI:** 10.1016/j.xagr.2026.100641

**Published:** 2026-04-06

**Authors:** Hamda Jama Yousuf, Mohamed Hussein Egeh, Ahmed Abdirahman Farah, Ali Ahmed Hussein, Abdiasis Aden Omer

**Affiliations:** Ogaansho Research and Consultancy Center, Burao, Somaliland

**Keywords:** immunization, Somaliland, Somaliland Demographic Health Survey, tetanus toxoid, zero-inflated negative binomial

## Abstract

**Background:**

Tetanus toxoid (TT) immunization is a critical public health intervention for reducing maternal and neonatal mortality. Despite global recommendations, TT coverage remains low in several low- and middle-income countries, including Somaliland.

**Objective:**

This study aims to assess the prevalence and determinants of TT immunization among pregnant women in Somaliland using the 2020 Somaliland Demographic Health Survey.

**Methods:**

A cross-sectional analytic study was conducted using the 2020 Somaliland Demographic Health Survey. Data from 2584 women aged 15 to 49 years. STATA 17 was used for analysis. Descriptive statistics were used to examine immunization coverage. The chi-square test was used to identify bivariate associations between TT uptake and explanatory variables. Given the nature of the count outcome and high proportion of zeros, a zero-inflated negative binomial regression model was applied. The result was interpreted using incidence rate ratios with 95% confidence intervals. The model with the lowest Akaike Information Criterion and Bayesian Information Criterion values and the highest log likelihood was selected as the best fit.

**Results:**

Only 14.24% of pregnant women received at least two TT doses, while 73.03% received none. Antenatal care (ANC) attendance (IRR=3.93; 95% CI: 3.32–4.66), wealth index, maternal employment (IRR=0.41; 95% CI: 0.23–0.75 for unemployed), institutional delivery, and distance to health facility (IRR=1.17; 95% CI: 1.02–1.34) were significantly associated with TT uptake. Regional disparities and media exposure were associated with a slightly lower rate of TT uptake.

**Conclusion:**

TT immunization among pregnant women in Somaliland is alarmingly low and shaped by both socio-demographic and reproductive health factors. Targeted interventions should focus on expanding ANC coverage, reducing geographic and economic barriers, empowering women, enhancing awareness through media, and improving physical access to healthcare facilities. These findings are aligned with Sustainable Development Goal 3 in Somaliland.

**AJOG Global Reports at a Glance**

**Why was this study conducted?**

Tetanus toxoid immunization during pregnancy is essential for preventing maternal and neonatal tetanus. However, coverage remains suboptimal in many low-resource settings, including Somaliland. This study aimed to identify determinants associated with poor tetanus toxoid immunization among pregnant women using nationally representative data.

**Key findings**

Several socio-demographic and healthcare-related factors were associated with low uptake of tetanus toxoid vaccination. Limited access to antenatal care, educational status, and socioeconomic factors significantly influenced immunization coverage among pregnant women.

**What does this add to what is known?**

This study provides national-level evidence from Somaliland using a zero-inflated negative binomial regression model, offering deeper insight into structural barriers affecting tetanus toxoid immunization and highlighting priority areas for targeted maternal health interventions.

## Background

Tetanus is a potentially fatal disease caused by Clostridium tetani, a bacterium whose spores are found worldwide.^1^ Tetanus is more prevalent in areas with poor sanitation, poverty, and limited access to healthcare.^2^ While tetanus can affect anyone, newborns and recently pregnant women are particularly vulnerable, especially if deliveries occur in an unsanitary environment.^3^ Unhygienic childbirth practices and a lack of maternal tetanus vaccination significantly elevate an infant’s risk. Despite being preventable, NT persists in many low- and middle-income countries, where its near-certain mortality rate is due to a lack of access to specialized medical care.^4^ When a pregnant woman is fully vaccinated against tetanus, she transfers protective antibodies to her baby via the placenta. This shields the infant from tetanus until they are old enough to receive their own vaccinations at 6 weeks.^5^

Worldwide, tetanus caused 38,000 deaths in 2017.^6^ Maternal and neonatal tetanus can be prevented by practicing good hygiene during childbirth and ensuring mothers are vaccinated against tetanus.^7^^,^^8^ This preventative approach is supported by research demonstrating that vaccinating women of childbearing age against tetanus lowers the risk of neonatal tetanus.^9^ Therefore, the World Health Organization advises that women with no prior tetanus vaccination receive a total of five doses of the tetanus toxoid (TT) vaccine. The initial two doses should be administered 1 month apart during the first trimester, followed by another dose during a subsequent pregnancy or within a year.^10^

In 2017, Sub-Saharan Africa and South Asia experienced the highest incidence of new tetanus cases, accounting for 82% of all cases globally. These regions also accounted for 29,500 tetanus-related deaths, representing 77% of the total mortality. Sub-Saharan Africa continues to have the highest rate of tetanus cases worldwide.^11^^,^^12^ Sub-Saharan African countries have alarmingly high tetanus mortality rates. For example, reports indicate mortality rates of 64% in Nigeria, 47% in Uganda, 43.1% in Tanzania, and 48.5% in Cameroon.^13^ Somalia, South Sudan, Afghanistan, and Kenya reported over 1000 newborn tetanus deaths per 100,000 people, indicating the highest prevalence of the disease. Somalia, South Sudan, and Kenya also had the highest rates of tetanus mortality after the newborn period (greater than five deaths per 100,000 people).^14^

In Somaliland, high maternal and neonatal mortality rates persist, with the 2020 Somaliland Demographic and Health Survey (SLHDS) showing low coverage of TT immunization among pregnant women.^15^ Before the 2020 SLHDS, available evidence indicated persistently low coverage of TT immunization in many low-resource settings, particularly in Sub-Saharan Africa.^13^^,^^14^ Earlier global estimates also highlighted a substantial burden of maternal and neonatal tetanus, reflecting gaps in access to immunization and maternal healthcare services.^7^^,^^8^ Over time, international and national efforts, including maternal immunization programs and expanded antenatal care (ANC) services, have aimed to improve coverage and reduce tetanus-related mortality.^12^ However, despite these efforts, recent data from the 2020 SLHDS indicate that a considerable proportion of pregnant women in Somaliland still do not receive the recommended doses of TT vaccination.^17^ This comparison highlights that, although progress has been made, significant gaps remain, suggesting the need for strengthened health system interventions and sustained policy commitment to improve maternal immunization coverage.

The SLHDS 2020 highlights that a significant proportion of pregnant women do not receive the recommended doses of TT vaccines. Research from around the world indicates that maternal age,^16^ education level,^17^ marital status,^18^ household wealth,^19^ distance to healthcare facilities,^20^ inadequate ANC,^21^ number of previous pregnancies,^22^ and the type of residence,^23^ where significantly associated with tetanus immunization before birth.

This study aims to provide reliable evidence that can inform policy and strategies to improve maternal and neonatal health outcomes in Somaliland. This research also contributes to the achievement of several Sustainable Development Goals (SDGs). It supports SDG 3, which aims to ensure healthy lives and promote well-being for all, particularly Targets 3.1 and 3.2 on reducing maternal and neonatal mortality. It also aligns with SDG 3.8 on universal health coverage. However, using the 2020 SLHDS dataset ensures a nationally representative sample, increasing the reliability of the findings. Addressing these key factors was essential for improving maternal and neonatal health outcomes in Somaliland.

## Method and material

### Study setting

The study was conducted in Somaliland, a self-declared independent country in the Horn of Africa with a population of approximately 4.5 million. Geographically, Somaliland is situated between Djibouti, Ethiopia, and Somalia, encompassing 176,119.2 km2, including an 850 km coastline, and featuring diverse terrain from coastal plains to hilly northern regions (1800–2100 m). The climate alternates between wet and dry seasons. The population is predominantly ethnic Somali and Muslim. Hargeisa, the capital, is home to over one-third of the population. Somaliland is administratively structured into six regions and 22 districts. The economy, though expanding due to livestock exports, faces challenges as Somaliland remains a low-income country with limited foreign investment and trade opportunities. Remittances and livestock exports to the Gulf States contributed to an estimated GDP of USD 2.5 billion and a per capita GDP of USD 566 in 2018. However, the absence of international recognition significantly restricts the nation’s access to bilateral agreements and direct donor investments.^15^^,^^24^

### Study design, data source, and study period

The research utilized data from the 2020 Somaliland Demographic Health Survey (SLHDS), the first nationally representative health survey in the country. This cross-sectional survey was conducted from August 2018 to December 2019. This design is appropriate for exploring the Determinants of Poor TT Immunization Among Pregnant Women in Somaliland. The 2020 SLHDS aimed to strengthen data systems and promote evidence-based planning within Somaliland.^15^

### Sample size and sampling

This study analyzed a weighted sample of 2584 women of reproductive age (15–49 years) from the 2020 Somaliland Demographic Health Survey (SLHDS) dataset. These women had experienced a pregnancy in the 5 years prior to the survey. The SLHDS utilized a two-stage sampling technique. First, a Geographic Information System was employed to map residential buildings and create enumeration areas (EAs), with each EA containing a minimum of 50 and a maximum of 149 dwellings. A total of 2923 EAs (1869 urban and 1054 rural), also known as primary sampling units (PSUs), were digitized. The final sampling frame comprised 2086 PSUs, accounting for security issues that prevented visits to some EAs. In the first stage of sampling, 35 EAs were selected from each region (Somaliland’s six first-level administrative divisions) using probability proportional to size sampling. A complete household listing was then conducted within each of the 35 selected EAs to determine the total number of households. Sample ground verification was also performed to refine the sampling frame as necessary.^15^

### Variables of the study

#### Outcome variable

The outcome variable in this study was the number of TT injections received before birth. Data were obtained from the 2020 SLHDS, which asked women whether they had received a TT injection during pregnancy. The response was recorded as a count (0, 1, 2, 3, 4, 5+) and analyzed as a count outcome variable to reflect the extent of immunization coverage.

#### Independent variables

The independent variables comprised a total of 19 explanatory factors, selected based on their theoretical relevance and prior research evidence. These consisted of both sociodemographic and reproductive health variables, socio demographic variables included sex of household head, Maternal age, Maternal education, husband’s education, Husband employment, maternal employment, place of residence, region, media exposure, wealth index, Reproductive health variables comprised Age of respondent at 1st birth, number of ANC visit, ANC utilization was categorized as not utilized (0 visits), partially utilized (1–3 visits), and adequately utilized (≥4 visits). Parity, distance to the health facility, place of birth, and healthcare decision-making.

#### Data processing and analysis

This study used Stata version 17 for data processing and analysis. First, the data was cleaned to remove errors like missing information, unusual values, and duplicates. Observations with missing values were excluded from the final analysis. Important factors such as socioeconomic background, access to healthcare, and TT immunization status were identified and prepared for analysis. Descriptive statistics (frequencies, percentages, averages, and standard deviations) were used to describe the study population. Then, chi-square tests were used to investigate the relationship between TT immunization and other factors. The main analysis involved a zero-inflated negative binomial (ZINB) regression model in Stata 17, which is good for data with a lot of zeros and over-dispersion than expected. This model helped us understand how factors like socioeconomic status, healthcare access, and education level are related to low immunization rates. Finally, determinants significantly associated with poor TT immunization were identified based on the Adjusted Incidence Rate Ratios and *P* values less than 0.05 in the final multivariable model. Model fit was assessed using the Akaike Information Criterion (AIC) and Bayesian Information Criterion (BIC), where lower values indicate better model performance and help guide model selection. Compared to other options, we’ll perform a sensitivity analysis to make sure the results are reliable. Finally, interpret the findings in light of existing research and suggest policy changes to improve immunization rates among pregnant women in Somaliland.

### Statistical method

Count data was the variable of interest in this study. Nonlinear models based on non-normal distributions are suitable for describing the relationship between a set of predictor variables and the response variable when the dependent variable is a count. The Poisson, NB regression, Zero-Inflated Poisson (ZIP), and ZINB models are the main frameworks used to model the relationship between the outcome variable and explanatory factors for count data.^25^

Unlike TT2+ indicators, count data captures the full distribution of TT doses received by pregnant women and allows for a more comprehensive assessment of immunization coverage. In addition, the presence of excess zero counts in the outcome variable justified the use of a ZINB model, which simultaneously models the frequency of doses and the likelihood of receiving no doses. This approach provides more detailed insights compared to binary indicators commonly used in previous studies.

### Poisson regression model

Poisson regression has been widely used for fitting count data. It is traditionally conceived as the basic count model upon which a variety of other count models are based.^26^ The Poisson probability mass function, with rate parameter *μ_i_*, is given by:

$$P\left(Y_{i}=y_{i}\right)=\frac{e^{-\mu_{i}}\;\mu_{i}^{y_{i}}}{y_{i}!},\;y_{i}=0,1,2,3,4,5\ldots ..$$

The expected count *μ_i_* is linked to explanatory variables through a log-linear function:

$$\log\left(\mu_{i}\right)=\beta_{0}+\beta_{1}x_{1}+\beta_{2}x_{2}+\beta_{3}x_{3}+\cdots +\beta_{p}x_{p}=X_{i}^{T}$$

where *X_i_* represents the vector of covariates (such as maternal age, education, residence, ANC visits), and *β* denotes the corresponding regression coefficients. A key assumption of the Poisson model is equidispersion, meaning the variance equals the mean:

E(Yi)=Var(Yi)=μi

Here, *β* is the vector of regression coefficients, and *X_i_* is the vector of covariates for the *i*th observation. However, in many real-world datasets, including this study, the variance exceeds the mean, a phenomenon known as overdispersion. Overdispersion can arise from omitted covariates, model misspecification, or excess zeros.^27^ In such cases, the Poisson model underestimates standard errors, leading to misleading inference.

### NB regression model

To address overdispersion, the NB regression model introduces a dispersion parameter *ϕ*>0. The NB probability mass function is:

$$P\left(Y_{i}=y_{i}\right)=\frac{\Gamma \left(y_{i}+\phi^{-1}\right)}{\Gamma \left(y_{i}+1\right)\;\Gamma \left(\phi^{-1}\right)}{\left(\frac{\phi^{-1}}{\phi^{-1}+\mu_{i}}\right)}^{\phi^{-1}}{\left(\frac{\mu_{i}}{\phi^{-1}+\mu_{i}}\right)}^{y_{i}},\;y_{i}=0,1,2,3,4,5..$$

The mean and variance are:

E(Yi)=μi,Var(Yi)=μi(1+ϕμi)

The mean and variance of the NB distribution are *E* [*y*|*μ ∅*]=*μ* and *V* [*y*|*μ ∅*]=*μ* (1 + *∅ μ*). Where *∅* is the dispersion parameter (if *∅*>0 and *μ*>0)? Special cases of the NB include the Poisson (*∅*=0) and the geometric (*∅*=1). The method of maximum likelihood is used to estimate the parameters in the NB regression model.^28^

### Zero-inflated regression model

Poisson regression and NB model with many zero outcomes on the response variable. The ZIP regression model is more effective for many zero outcomes than Poisson regression. While the ZINB regression model is more effective for many zero outcomes than the NB regression.^29^

### ZIP and ZINB regression models

In ZIP regression, the counts *Y_i_* equal 0 with probability *p_i_* and follow a Poisson distribution with mean *µ_i_*, with probability 1−*p_i_*, where *i*=0, 1, 2… *n*. The ZIP model can thus be seen as a mixture of two-component distributions, zero parts, and nonzero components, given by.^26^

$$P\left(Y_{i}=0\right)=\pi_{i}+\left(1-\pi_{i}\right)e^{-\mu_{i}}$$

$$P\left(Y_{i}=y_{i}\right)=\left(1-\pi_{i}\right)\frac{e^{-\mu_{i}}\mu_{i}^{y_{i}}}{y_{i}!},\;y_{i}>0$$

The ZINB distribution is a mixture distribution assigning a mass of *p* to “extra” zeros and a mass of (1−*p*) to an NB distribution, where 0≤*p*≤1. Based on the probability function of the zero–modified distribution, the probability mass function for ZINB is:

$$P\left(Y_{i}=0\right)=\pi_{i}+\left(1-\pi_{i}\right){\left(\frac{\phi^{-1}}{\phi^{-1}+\mu_{i}}\right)}^{\phi^{-1}}$$

$$P\left(Y_{i}=y_{i}\right)=\left(1-\pi_{i}\right)\cdot \frac{\Gamma \left(y_{i}+\phi^{-1}\right)}{\Gamma \left(y_{i}+1\right)\;\Gamma \left(\phi^{-1}\right)}\;{\left(\frac{\phi^{-1}}{\phi^{-1}+\mu_{i}}\right)}^{\phi^{-1}}{\left(\frac{\mu_{i}}{\phi^{-1}+\mu_{i}}\right)}^{y_{i}},\;y_{i}>0$$

Given the nature of TT immunization data from the SLHDS 2020, which likely exhibits overdispersion and excess zeros, this study compares the performance of Poisson, NB, ZIP, and ZINB models to identify the best fit for describing the determinants of TT immunization counts among pregnant women in Somaliland. Maximum likelihood estimation is used to fit these models, and model comparison is based on criteria such as AIC and likelihood ratio tests. This approach ensures robust inference on socio-demographic and healthcare factors influencing TT immunization uptake.

## Results

Table 1 presents the distribution of TT injections received before birth among pregnant women in Somaliland based on the SLHDS 2020 data. The findings indicate that a substantial proportion of the respondents (73.03%) did not receive any TT injections during pregnancy. Only 12.73% received one dose, while a relatively small percentage of women received the recommended two or more doses required for adequate protection against neonatal tetanus. Specifically, 7.93% received two doses, 3.79% received three doses, 0.43% received four doses, and 2.09% received five or more doses. The mean number of TT injections was 0.52, with a standard deviation of 1.05, suggesting limited uptake and some variability in vaccination coverage. The number of doses ranged from 0 to 5, with the majority falling at the lower end of the scale. These results highlight a concerning gap in maternal immunization coverage, as only 14.24% of pregnant women received at least two doses, which is the minimum required for effective immunological protection. This underscores the urgent need for targeted public health interventions to enhance access to and utilization of maternal tetanus immunization services in Somaliland. The mean and the variance of the number of TT injections before birth was 0.52 and 1.11, respectively (Table 1).

**Table 1:** Number of tetanus toxoid injections before birth and associated factors among pregnant women in Somaliland, SLHDS2020

Number of tetanus toxoid injections before birth	Frequency	Percentage
0	1887	73.03
1	329	12.73
2	205	7.93
3	98	3.79
4	11	0.43
5+	54	2.09
Mean	0.5212848	
SD	1.051303	
Variance	1.105238	
Minimum	0	
Maximum	5	
Total observation	2584	

Table 2 presents the socio-demographic and reproductive health characteristics of pregnant women in Somaliland based on the SLHDS 2020 dataset. The largest proportion of respondents was aged 25 to 29 years (27.55%), followed by those aged 30 to 34 years (21.17%) and 20 to 24 years (17.92%). The majority of the women resided in either Sanaag (23.03%) or Sool (22.87%), with nearly equal representation from rural (49.61%) and urban (50.39%) areas. A substantial proportion of respondents were in the lowest wealth quintile (39.59%). Alarmingly, 83.55% of the women had no formal education, and only 1.04% attained higher education. Most husbands were unemployed (61.42%), and almost all women were not employed (99.69%). Additionally, 62.42% of households were headed by males. Regarding husbands’ education, 90.56% had no formal education. Media exposure was limited, with only 35.49% of women reporting access. Nearly half (47.52%) of the respondents gave birth for the first time before the age of 20. Decision-making about healthcare was most commonly made by the husband alone (40.56%), followed by joint decision-making (38.85%). A significant number (67.41%) reported that distance to health facilities was a major problem. ANC utilization was low, with 63.43% not attending any visits, and only 12.93% receiving adequate care. Furthermore, 73.45% of the women gave birth at home. Regarding parity, the majority were either grand multiparous (44.78%) or multiparous (43.30%), with only 11.92% being primiparous.

**Table 2:** Characteristics of women who participated in the study assessing tetanus toxoid immunization among pregnant women in Somaliland (2584) SLHDS2020

Variables	Categorize	Frequency	Percentage
Age	15–19	134	5.19
	20–24	463	17.92
	25–29	712	27.55
	30–34	547	21.17
	35–39	464	17.96
	40–44	202	7.82
	45–49	62	2.40
Region	Awdal	347	13.43
	Marodijeh	300	11.61
	Sahil	312	12.07
	Togdheer	439	16.99
	Sool	591	22.87
	Sanaag	595	23.03
Place of residence	Rural	1282	49.61
	Urban	1302	50.39
Wealth index	Lowest	1023	39.59
	Second	428	16.56
	Middle	266	10.29
	Fourth	396	15.33
	Highest	471	18.23
Maternal educational status	No education	2159	83.55
	Primary	337	13.04
	Secondary	61	2.36
	Higher	27	1.04
Husband employment	Yes	997	38.58
	No	1587	61.42
Maternal employment	Yes	8	0.31
	No	2576	99.69
Sex of household head	Male	1613	62.42
	Female	971	37.58
Husband’s educational status	No	2340	90.56
	Yes	244	9.44
Media exposure	No	1667	64.51
	Yes	917	35.49
Age of respondent at 1st birth	<20	1228	47.52
	20–24	963	37.27
	25–30	341	13.20
	30 and above	52	2.01
Health care decision making	Respondent	525	20.32
	Husband	1048	40.56
	Respondent and husband jointly	1004	38,85
	In-laws	3	0.12
	Someone else	2	0.08
	Other	2	0.08
Distance from the health facility	Big problem	1742	67.41
	Not a big problem	842	32.59
Number of ANC visits	Not utilized	1639	63.43
	Partially utilized	611	23.65
	Adequately utilized	334	12.93
Place of birth	Home	1898	73.45
	Health institution	686	26.55
Parity	Primipara	308	11.92
	Multipara	1119	43.30
	grand multipara	1157	44.78

Table 3 presents the comparison of four count regression models—Poisson, NB, ZIP, and ZINB—used to analyze the number of TT injections received by pregnant women in Somaliland, based on SLHDS 2020 data. Initially, the standard Poisson regression model was considered. However, this model assumes that the mean and variance of the count data are equal, an assumption that was violated in the dataset due to evident over-dispersion and excess zeros. The data characteristics suggested unobserved heterogeneity and a high frequency of women reporting zero doses of TT, which necessitated the use of more flexible models. To address the over-dispersion, the NB model was tested, as it incorporates a dispersion parameter that allows the variance to exceed the mean. To further account for the large number of zero responses, the ZIP and ZINB models were also evaluated. These models assume a dual process: one generating true counts (including positive counts) and another generating excess zeros, making them especially suitable for datasets with structural zeros. Model performance was assessed using standard model fit criteria: AIC, BIC, and log-likelihood values. Lower AIC and BIC values indicate a better model fit, while a higher log-likelihood indicates a more likely model given the observed data. Among the models tested, the ZINB model exhibited the best fit, with the lowest AIC (2160.776) and BIC (2389.203), as well as the highest log-likelihood value (−1041.388). These results suggest that the ZINB model outperformed the other models in accounting for both the over-dispersion and excess zeros present in the data. Consequently, the ZINB model was selected as the most appropriate model for analyzing the number of TT injections received before birth among pregnant women in Somaliland. This model provided the best balance between

**Table 3:** Model comparison for the number of tetanus injections before birth and associated factors among pregnant women in Somaliland, SLHDS2020

Models				
Test statistics	Poisson	NB	ZIP	**ZINB**
AIC	4931.374	4581.706	2163.009	**2160.776**
BIC	5165.658	4821.847	2397.293	**2389.203**
Log likelihood	−2425.6871	−2249.8528	−1041.505	**−1041.388**

Bold values indicate statistically significant results at p < 0.05.

Table 4 shows the ZINB regression model identified several significant predictors of the number of TT injections received before birth among pregnant women in Somaliland using the SLHDS 2020 data, region, maternal employment, ANC utilization, household wealth index, media exposure, distance from a health facility, and place of birth showed significant associations with the frequency of tetanus injections before birth. The number of tetanus injections before birth was significantly lower among pregnant women living in Marodijeh (IRR=0.68; 95% CI: 0.55–0.84), Sahil (IRR=0.71; 95% CI: 0.58–0.87), Togdheer (IRR=0.81; 95% CI: 0.68–0.98), and Sool (IRR=0.71; 95% CI: 0.58–0.86) compared to women residing in Awdal region. Pregnant women in higher wealth categories were significantly more likely to receive tetanus injections before birth than those in the lowest wealth category, with IRRs of 1.63 (95% CI: 1.36–1.97) for the second quintile, 1.76 (95% CI: 1.43–2.16) for the middle, 1.32 (95% CI: 1.08–1.62) for the fourth, and 1.54 (95% CI: 1.26–1.88) for the highest wealth quintile. The number of tetanus injections before birth was 0.41 times lower (IRR=0.41; 95% CI: 0.23–0.75) among unemployed women compared to employed women. Women who reported that distance to a health facility was not a big problem had a slightly higher frequency of tetanus injections (IRR=1.17; 95% CI: 1.02–1.34). Similarly, women who were exposed to media had a slightly lower rate of TT uptake (IRR=0.89; 95% CI: 0.78–1.00). ANC utilization was one of the strongest predictors; women who partially utilized ANC were 3.25 times (IRR=3.25; 95% CI: 2.82–3.76) more likely, and those who adequately utilized ANC were 3.93 times (IRR=3.93; 95% CI: 3.32–4.66) more likely to receive tetanus injections compared to those who did not utilize ANC at all. Lastly, women who gave birth in health institutions had a higher number of tetanus injections before birth (IRR=1.15; 95% CI: 1.00–1.31) compared to those who delivered at home.

**Table 4:** Count and zero-inflation components of the zero-inflated negative binomial regression model for tetanus toxoid injections among pregnant women in Somaliland, SLHDS 2020

Variables	Categorize	Zero-inflated negative binomial regression model	
		Count model (IRR, 95% CI)	Inflation model (OR, 95% CI)
Age	15–19	Ref	Ref
	20–24	1.08 (0.80–1.44)	
	25–29	1.06 (0.77–1.45)	
	30–34	1.08 (0.76–1.52)	
	35–39	1.14 (0.79–1.66)	
	40–44	1.06 (0.69–1.64)	
	45–49	1.08 (0.60–1.92)	
Region	Awdal	Ref	Ref
	Marodijeh	0.68 (0.55–0.84)^c^	
	Sahil	0.71 (0.58–0.87)^b^	
	Togdheer	0.81 (0.68–0.98)^a^	
	Sool	0.71 (0.58–0.86)^b^	
	Sanaag	0.99 (0.83–1.20)	
Place of residence	Rural	Ref	Ref
	Urban	1.11 (0.99–1.25)	1.43 (1.01–2.02)^a^
Maternal educational status	No Education	Ref	
	Primary	0.95 (0.82–1.10)	
	Secondary	1.04 (0.78–1.39)	
	Higher	1.01 (0.67–1.52)	
Wealth index	Lowest	Ref	Ref
	Second	1.63 (1.36–1.97)^c^	0.56 (0.35–0.90)^a^
	Middle	1.76 (1.43–2.16)^c^	0.33 (0.20–0.56)^c^
	Fourth	1.32 (1.08–1.62)^b^	0.42 (0.24–0.72)^b^
	Highest	1.54 (1.26–1.88)^c^	0.19 (0.11–0.34)^c^
Maternal employment	Yes	Ref	Ref
	No	0.41 (0.23–0.75)^b^	
Husband employment	Yes	Ref	Ref
	No	1.08 (0.95–1.23)	
Husband’s educational status	No	Ref	Ref
	Yes	1.10 (0.93–1.29)	
Sex of household head	Male	Ref	Ref
	Female	0.96 (0.86–1.08)	
Media exposure	No	Ref	Ref
	Yes	0.89 (0.78–1.00)^a^	1.28 (0.87–1.90)
Age of respondent at 1st birth	<20	Ref	Ref
	20–24	0.98 (0.86–1.13)	
	25–30	0.89 (0.72–1.11)	
	30 and above	1.01 (0.68–1.49)	
Health care decision making	Respondent	Ref	Ref
	Husband	0.95 (0.82–1.10)	
	Respondent and husband jointly	0.87 (0.75–1.00)	
	In-laws	(Omitted)	
	Someone else	1.05 (0.25–4.39)	
	Other	0.55 (0.07–4.08)	
Distance from the health facility	Big problem	Ref	Ref
	Not a big problem	1.17 (1.02–1.34)^a^	1.26 (0.87–1.82)
Number of ANC visits	Not utilized	Ref	Ref
	Partially utilized	3.25 (2.82–3.76)^c^	0.04 (0.02–0.07)^c^
	Adequately utilized	3.93 (3.32–4.66)^c^	**0.01 (0.00–0.09)**^c^
Place of birth	Home	Ref	Ref
	Health institution	1.15 (1.00–1.31)^a^	0.63 (0.40–0.99)^a^
Parity	Primipara	Ref	Ref
	Multipara	0.89 (0.73–1.08)	
	Grand multipara	0.94 (0.73–1.21)	

*CI*, confidence interval; *IRR*, incidence rate ratio; *OR*, odds ratio.

a *P*<.05

b *P*<.01

c *P*<.001.

The zero-inflation component of the ZINB model identified factors associated with the likelihood of receiving no TT doses. ANC utilization showed a strong protective effect against zero TT uptake, as women who partially utilized ANC (OR=0.04; 95% CI: 0.02–0.07) and those who adequately utilized ANC (OR=0.01; 95% CI: 0.00–0.09) had significantly lower odds of receiving no doses compared to those who did not utilize ANC.

Similarly, women from higher wealth categories were less likely to have zero TT doses, with progressively lower odds observed across increasing wealth quintiles. In contrast, urban residence was associated with a higher likelihood of zero TT uptake compared to rural residence (OR=1.43; 95% CI: 1.01–2.02). Additionally, women who delivered in health institutions had lower odds of having zero TT doses compared to those delivering at home (OR=0.63; 95% CI: 0.40–0.99). These findings highlight important structural and socioeconomic determinants of complete nonutilization of TT immunization.

## Discussion

### Principal findings

This study examined the factors associated with the number of TT injections received by pregnant women in Somaliland using data from the 2020 Somaliland Demographic Health Survey. The findings revealed a significant association between TT immunization and several socio-demographic and health care access factors. Only 14.24% of the respondents received at least two doses, with 73.03% receiving none.

### Results in the context of what is known

The findings are lower compared to the studies conducted in Africa, reported 62%^30^ And Brazil reported 59.2%,^31^ and Turkey reported 27.8%.^32^ The current finding also falls slightly below that of Sierra Leone, with coverage reported at 82.1%.^33^ The disparity may be due to differences in healthcare infrastructure, maternal education levels, and ANC utilization. Moreover, Somaliland’s limited healthcare and financing accessibility challenges may further explain the low TT uptake. One of the strongest predictors of TT immunization was the region of residence, with women in some regions, such as Maroodijex, Togdheer, Saahil, and Sool, being significantly less likely to receive the TT injection compared to those from Awdal. These regional differences reflect variations in the availability and quality of maternal health services across Somaliland. A similar pattern was reported in a study from Nigeria and Ghana, where geographic inequalities were found to influence access to maternal immunization and ANC.^34^

### Clinical implications

Women in remote areas often face difficulties such as a long distance to the health facilities and a lack of transportation, which limit their ability to receive essential services like TT immunization. Women from households in higher wealth quantiles were significantly more likely to receive TT immunization; for example, those in the middle wealth quantile had 1.76 times higher odds of obtaining the TT dose than women in the poorest category. These findings align with previous studies in Kenya,^21^ South Africa,^35^ Alexandra,^36^ and India,^20^ which indicate that economic status plays a crucial role in determining access to maternal health care services, including immunization. Financial constraints can hinder women’s ability to travel to health facilities or pay for services, thus reducing their likelihood of receiving recommended vaccines.^37^^,^^38^

### Research implications

The study also found that maternal employment status influences TT uptake. Unemployed women were 59% less likely to receive the TT immunization compared to those employed. Employment may empower women economically and socially, enabling them to make health-related decisions and access services independently. This is supported by research suggesting that working women are more likely to utilize maternal health services due to increased autonomy and financial resources.^21^^,^^39^ In contrast, media exposure was associated with a slightly lower rate of TT uptake (IRR=0.89; 95% CI: 0.78–1.00). This unexpected finding may suggest that media messaging alone is insufficient to translate into behavioral change in this context, or that other structural barriers limit the effectiveness of health information dissemination.^40^ Access to health facilities was also a key determinant; women who reported that distance was not a major problem were more likely to receive the TT immunization (IRR=1.17, *P*=.022). Long distances, poor roads, and a lack of transportation are known barriers in low-resource settings, especially in rural Somaliland, which shares similar challenges with regions in rural Ethiopia and northern Nigeria.^34^ Several barriers contribute to this issue; the expense and time involved in reaching far-off immunization centers are the primary factors. Their significant home responsibility, such as caring for children and managing the household, often keep them at home, making them reluctant to travel to distant clinics. Furthermore, the need for multiple vaccination visits, such as those for TT, can be exhausting for both women and their children if these centers are not conveniently located.^41^

One of the most striking findings was a strong association between ANC attendance and TT uptake. Compared to those who did not attend ANC, women who partially utilized ANC were 3.25 times more likely to receive TT, while those who adequately utilized ANC were 3.93 times more likely. This was consistent with studies reported in Kenya.,^21^ Sierra Leon,^33^ Ghana,^42^ Mali,^43^ Malwa,^44^ Pakistan,^45^ and Turkey.^46^ This confirms the critical role of ANC as an entry point for delivering maternal vaccines, as emphasized in global health recommendations. The low ANC utilization in this study (63.43% had no ANC visit) represents a significant missed opportunity for improving TT coverage and maternal health more broadly. The Place of delivery also had a modest but notable association with TT coverage. Women who gave birth in a health institution were 15% more likely to be immunized than those who delivered at home. This may be due to increased health education, follow-up visits, and postpartum counseling typically provided in a facility setting, as well as institutional promotion of immunization schedules.

#### Policy implications

These findings have important policy implications. Strengthening ANC services should be a priority, as ANC utilization was the strongest predictor of TT uptake. Expanding access to maternal health services, particularly in underserved regions, and reducing financial and geographic barriers to care are essential to improve immunization coverage. In addition, targeted health education and outreach programs should be implemented to increase awareness and promote the importance of maternal vaccination. Policymakers should also focus on addressing socioeconomic inequalities to ensure equitable access to maternal healthcare services.

### Strengths and limitations

A major strength of this study is the use of nationally representative data from the 2020 SLHDS, which enhances the generalizability of the findings to pregnant women across Somaliland. The large sample size and standardized data collection procedures improve the reliability and comparability of the results. In addition, the application of a ZINB regression model is a methodological strength, as it appropriately accounts for over-dispersion and excess zeros in the count outcome of TT injections.

However, the study has some limitations. The cross-sectional design limits the ability to establish association relationships between explanatory factors and TT immunization uptake. The reliance on self-reported vaccination information may introduce recall bias or misclassification. Furthermore, some potentially important factors, such as vaccine availability at health facilities, quality of ANC services, and provider-level practices, could not be assessed due to data limitations. Despite these limitations, the study provides valuable population-level evidence on the determinants of TT immunization among pregnant women in Somaliland.

## Conclusion

The study concludes that TT immunization coverage among pregnant women in Somaliland is alarmingly low and far below the recommended global targets. The number of TT doses received is strongly influenced by both individual and structural factors. Women who resided in certain regions, such as Marodijeh, Sahil, Togdheer, and Sool, were significantly less likely to receive TT immunization. Indicating geographic disparities in the availability and access. Similarly, women from higher wealth quantiles were more likely to be immunized, suggesting that financial capacity enhances health care utilization. Employment status was also influential, with Employed women more likely to be vaccinated, possibly due to increased autonomy and resources. Physical access to health facilities and attendance of ANC visits were among the strongest predictors, with ANC providing an essential opportunity for administering the TT vaccine. Women who gave birth in a health institution also had a higher immunization rate, showing that facility-based care contributes positively to maternal health practice.

## CRediT authorship contribution statement

**Hamda Jama Yousuf:** Supervision, Software, Formal analysis, Data curation, Conceptualization. **Mohamed Hussein Egeh:** Formal analysis. **Ahmed Abdirahman Farah:** Conceptualization. **Ali Ahmed Hussein:** Writing – original draft, Visualization. **Abdiasis Aden Omer:** Methodology.
